# Evaluation of methods for detection of fluorescence labeled subcellular objects in microscope images

**DOI:** 10.1186/1471-2105-11-248

**Published:** 2010-05-13

**Authors:** Pekka Ruusuvuori, Tarmo Äijö, Sharif Chowdhury, Cecilia Garmendia-Torres, Jyrki Selinummi, Mirko Birbaumer, Aimée M Dudley, Lucas Pelkmans, Olli Yli-Harja

**Affiliations:** 1 Department of Signal Processing, Tampere University of Technology, P.O.Box 553, Tampere, 33101, Finland; 2 Institute for Systems Biology, 1441 N. 34th Street, Seattle, WA, 98103-8904, USA; 3 Institute of Molecular Systems Biology, ETH Zürich, Wolfgang-Pauli-Str. 16, Zürich, 8093, Switzerland

## Abstract

**Background:**

Several algorithms have been proposed for detecting fluorescently labeled subcellular objects in microscope images. Many of these algorithms have been designed for specific tasks and validated with limited image data. But despite the potential of using extensive comparisons between algorithms to provide useful information to guide method selection and thus more accurate results, relatively few studies have been performed.

**Results:**

To better understand algorithm performance under different conditions, we have carried out a comparative study including eleven spot detection or segmentation algorithms from various application fields. We used microscope images from well plate experiments with a human osteosarcoma cell line and frames from image stacks of yeast cells in different focal planes. These experimentally derived images permit a comparison of method performance in realistic situations where the number of objects varies within image set. We also used simulated microscope images in order to compare the methods and validate them against a ground truth reference result. Our study finds major differences in the performance of different algorithms, in terms of both object counts and segmentation accuracies.

**Conclusions:**

These results suggest that the selection of detection algorithms for image based screens should be done carefully and take into account different conditions, such as the possibility of acquiring empty images or images with very few spots. Our inclusion of methods that have not been used before in this context broadens the set of available detection methods and compares them against the current state-of-the-art methods for subcellular particle detection.

## Background

Recent advances in cell imaging technologies include accurate stage controllers, improved optics, increased camera resolution, and, perhaps most importantly, fluorescent staining of specific cellular components. Together these advances enable automated image acquisition of small subcellular objects with the goal of providing insight into phenotypes and cellular functions [1-4]. With increased imaging throughput and large-scale data acquisition, the challenge of image interpretation and information extraction has also shifted from visual inspection or interactive analysis to more automated methods [5,6].

Accurate and automated subcellular object segmentation is essential for a variety of applications. For example, interpreting complex cellular phenotypes is typically dependent on identifying and quantifying various parameters associated with small organelles, setting high requirements for the accuracy of the image analysis [7]. Also the analysis of cellular structures based on 3D images obtained with fluorescence and confocal microscopes requires accurate detection. Advances in such methods will improve our ability to model small organelles in 3D [8]. Further, live-cell imaging with specific molecular probes has brought image tracking to subcellular level, and thus reliable object detection over the course of the imaging period adds a temporal dimension to image analysis [9,10].

A variety of subcellular object detection methods have been described in the literature (examples are listed in Table 1). Due to the specific applications they have been designed for, the algorithms are usually very problem-specific. However, it is rare to see choice of a detection method based on experimental thorough testing under a variety of conditions or comparisons against other previously proposed spot detection methods. Rather, it is still common to use naïve comparisons of particle detection algorithms against histogram thresholding methods applied on intensity information. For example, Otsu's thresholding [11], which seeks to maximize between-class variance, is widely applied as a reference method. However, for the segmentation of small spots in the presence of relatively high background fluorescence global thresholding approaches usually fail. Thus, comparative studies of the performance of subcellular object detection methods under a variety of different conditions are needed.

**Table 1:** Summary of methods.

Algorithm	Description	Free parameters
Band-pass filtering (BPF)	Object intensity enhancement with bandpass FIR filtering	4
Feature point detection (FPD) [9]	Percentile detection with non-particle discrimination	3
h-dome detection (HD) [16]	h-dome morphological filtering	5
Kernel methods (KDE) [21]	Kernel density estimation with a family of kernels	3
Local comparison (LC)	Maximization between direction-specific image convolutions	2
Locally enhancing filtering (LEF)	Local signal enhancement and background suppression	1
Morphometry (MGI) [23]	Morphometry with granulometric analysis	0
Multiscale wavelets (MW) [26]	Multiscale product of wavelet coefficients	2
Source Extractor (SE) [27]	Convolution applied for background clipped image	4
Sub-pixel localization (SPL) [10]	Fitting of Gaussian kernels to local intensity maxima	1
Top-hat filtering (THE) [29]	Top-hat filtering and entropy-based thresholding	1

Summary of methods, with method abbreviation used in this study and short description of main principle. The number of free parameters refers to the parameters that were tuned when optimizing the methods for the image sets.

Evaluating the performance of image segmentation algorithms has been a long-standing challenge. Validating segmentation results usually requires a ground-truth reference, and in biomedical applications the task of generating such reference falls to an expert biologist. This burdensome and error-prone strategy becomes even more challenging when evaluating small, but numerous subcellular organelles, particularly in the context of high-throughput experiments. In these cases, common limitations in the focus, contrast and resolution of the images render reliable pixel-level outlining of objects nearly impossible. Alternative evaluation methods include the use of computer-generated images for direct comparisons to ground truth results, experimentally derived control vs. test samples, and evaluations that measure performance as a function of an input stimulus that enable indirect comparisons between different conditions. Recently, benchmark image collections of cells and other types of biological samples have been developed to facilitate comparison and validation of image analysis methods [12-14].

In this study, we compare the performance of several algorithms for finding subcellular objects (i.e. small, bright spots) in fluorescence microscopy images. The algorithms employ various approaches for segmenting small structures, all aimed at detecting spot-like local intensity peaks, as opposed to the general separation of signal from background that is common in cell segmentation. We also propose an objective and comprehensive approach for evaluating algorithms for small particle detection. We use indirect comparisons with high-throughput well plate data, comparisons against manually scored objects in frames of 3D image stacks, and pixel-level comparisons against ground truth results in simulated images.

Importantly, our comparison study takes into account various situations, such as cases where a part of spots are severely blurred, emulating the typical situations of out-of-focus and diffraction limited appearance. Our comparison also considered cell heterogeneity (in this case images with varying number of spots), a factor commonly encountered in high throughput screening assays. In such case, the detection algorithms must be able to cope both with a range of conditions, such as cells ranging from low to high spot concentration in cells. Especially in high-throughput settings, tuning of parameters needs to be done for the whole screen, not for individual images.

Recently, a comparative study of nine commonly used spot detection methods has been published [15,16]. Here, we expand the set of methods evaluated while also taking into account the results in [15,16] by including the top-performing unsupervised method in our study. Further, our study covers a wide set of usage scenarios by applying three different image sets, providing a set of methods tested in various conditions, including methods that have not been used in the context of subcellular object detection before. The set of methods serves also as a resource for developers of novel particle detection algorithms, enabling more reasonable and informative comparison than histogram thresholding of intensity values.

## Methods

### Methods for detecting subcellular objects

A set of eleven algorithms covering a wide range of techniques for spot detection was selected for this study. Our selection includes eight previously published methods that were initially developed for applications other than subcellular spot detection. In addition, we formulate three filtering-based methods that, to the best of our knowledge, have not been previously applied to subcellular object detection. The detection of small subcellular particles from images can be divided into three phases [16]: First, an optional preprocessing phase can be used to reduce noise and to attenuate objects of a desired shape or size. Due to limitations in imaging technology, an accurate representation of the biological sample can be degraded by several error sources, resulting in a noisy observation of the underlying object. To decrease the effect of these errors, an optional low-pass filtering phase for noise suppression can be applied, and here the linear low pass filtering has been applied depending on whether the method has been observed to suffer from false detections due to background noise and the choice has been made through testing separately for each image set and method. We leave, however, experimenting with various preprocessing methods out of the scope of this article. Next, signal enhancement may be used to make the desired objects more easily detectable than they were in the original image. Many of the methods studied here involve user-definable parameters for controlling this phase. We use grid-search for tuning such parameters (described below). Finally, the actual detection is obtained by thresholding the enhanced signal. Because we do not consider segmentation threshold as a parameter for the detection methods unless it has been defined as such in method description, the presented methods derive the detection result automatically based on heuristics rather than stepping through multiple threshold levels. As a result, our comparison shows the results as given by the methods after tuning their parameters in a grid-search manner, not after fixing the operation point by tuning the segmentation threshold. In this way, the methods can be compared based on their performance when operating in a fully automated manner.

The large number and wide variety of methods designed for intensity detection in different image analysis applications preclude an analysis of all possible methods described in the literature. We chose to exclude methods relying on statistical learning, such as in [17], to avoid the problem of selecting training data. We also left of methods relying on pure intensity thresholding, since they are are likely to perform poorly due to non-uniform background and staining in the cell bodies. However, our selection does cover a variety of different approaches, which are relatively comparable in terms of accuracy and processing time. The selected algorithms are listed in Table 1 with a brief note on their operation principle and the abbreviations of their names used throughout the manuscript. Below is a more detailed description of each algorithm.

#### Band-pass filtering

In this method we formalize a detection method based on band-pass filtering (BPF). Here the image is band-pass filtered using a filter with transfer function *H *in a frequency domain that produces an image in which the objects of interest are emphasized. In addition to emphasizing the objects, the band-pass filter can be used to suppress the presence of noise, e.g. shot noise can be taken out by filtering the high-frequency components.

The filter *H *is designed such that the normalized cut-off frequencies are . Because the choice of cut-off frequencies is not a straightforward task from the spatial domain, it is advisable to consider the spectrum while choosing the desired band-passes. After filtering, Otsu's method [11] is used to automatically obtain a threshold value *th *for binarizing the band-pass filtered image. Thus, the four cut-off frequencies are the only user-defined variables.

#### Feature point detection

The feature point detection (FPD) algorithm proposed as a part of a tracking framework in [9] was originally designed for colloidal studies in [18]. The algorithm first reduces background effects in an image restoration step by box-car average estimation, and simultaneously enhances spot-like structures by convolving with a Gaussian kernel [9]. More formally, the convolution kernel is given as(1)

where  and *B *are normalization constants, *λ*_*n *_defines the Gaussian kernel width, and *w *is a user-tunable kernel window size [9]. Thus, the filtered image after the convolution with *K*^*w *^(*i*, *j*) is given as(2)

where *f*(*x*, *y*) is the original image, (*x*, *y*) and (*i*, *j*) are pixel coordinates in the image and kernel, respectively.

The initial point locations are then estimated by finding local intensity maxima. A point is considered to be a local maximum if it has the highest intensity within a local window, and the intensity value falls within the *r *highest percentile. The algorithm then proceeds by refining the point locations. Finally, all detected points are subjected to non-particle discrimination in the zeroth and second order intensity moment space, where a user-defined threshold *T*_*s *_controls the discrimination. A detailed description of the discrimination step can be found in [9]. Thus, the percentile threshold, the discrimination threshold, and the window size parameter (related to the particle size) are the three free parameters for FPD in this study. We note that one feature of the applied FPD implementation is that it was used for giving the object locations as an output instead of a segmentation result. As a result, detection of an object can be evaluated but direct comparison of the segmentation result is not done here.

#### h-dome transform

The morphological h-dome transform (HD) [19] has been applied to subcellular object detection in a tracking context [20]. Smal et al. [15,16] reported the best results among unsupervised object detectors were achieved with the h-dome transform based detector. The h-dome detector, according to [16], assumes that the image is formed by *N*_*o *_objects of interest, heterogeneous background structures and intensity distribution *B*(*i*, *j*), and a noise term *η*(*i*, *j*). The aim of the method is to estimate the number of objects *N*_*o *_and the object locations in image.

Briefly, the h-dome detection method as presented in [16] proceeds as follows. First, the input image *f*(*i*, *j*) is LoG filtered to obtain a background subtracted image , where spots are enhanced. Filtering is controlled by a parameter *σ*_*L *_which defines the scale. Next, a grayscale reconstruction of the filtered image is created using a mask (*i*, *j*) - *h*, where *h *> 0. Thus, the image decomposition is given as(3)

where *H*_*σ *_contains the small objects, the grayscale reconstruction *B*_*σ *_represents larger background structures, and intensities of height *h *are cut-off from the top. The h-dome transformed image *H*_*σ*_, where the bright objects should all have an intensity value of *h*, is used as a probability map for sampling and where pixel values of *H*_*σ *_are raised to the power of *s*. The map  reveals areas that are likely to contain spots. After sampling, the samples are divided into clusters using the mean-shift algorithm. Using the mean value and variance of each cluster, the samples are divided into real objects and other structures, while the parameter *σ*_*M *_controls the maximum allowed size for an object of interest. Details about sampling and object discrimination can be found in [16]. Overall the h-dome transformation based method has several parameters that need to be tuned based on the data in order to obtain useful results. As reported in [15,16], the method is a very powerful detector when the parameters are tuned reasonably and when the data supports the assumptions made by the model.

#### Kernel density estimation

A segmentation algorithm based on the use of kernel density estimation (KDE) is presented in detail in [21], this method is also known as the Parzen window method. Briefly, the method estimates the probability density function over the image by combining local information. The estimation step results in a smoothed version of the original image where the effect of noise is suppressed.

The method processes the image *f *by filtering it with a desired kernel in a circular window placed in coordinate (*i*, *j*) as follows:(4)

where *h *is the smoothing parameter, (*k*, *l*) denotes pixel coordinate inside kernel, card is the cardinality of the set, and the kernel *K*(*u*) could be, e.g., uniform *K*(*u*) = [|*u*| ≤ 1]. Other implemented kernels are Gaussian, Epanechnikov, triangle, quartic, triweight and cosine [22]. Finally, Otsu's method [11] is used to obtain a binarized version of the original image. In this method there are three parameters that can be set by the user: the radius *R*, the smoothing parameter *h *and the kernel. However, the choice of the kernel used is not crucial [21] to the result.

#### Local comparison and selection

**Figure 1:**
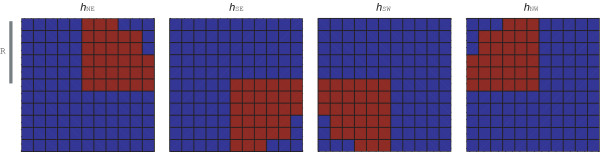
**Direction-specific filters *h*_*NE*_, *h*_*SE*_, *h*_*SW*_, and *h*_*NW *_for the local comparison method**. Red pixels show the area that is taken into account around each filtering position (*i*, *j*). Here the radius *R *is set to 5.

The local comparison and selection (LC) algorithm is a novel method for subcellular object detection. LC uses multiple spatial filters and performs comparison between their outputs. First, we start with a circular filter *h *of the radius *R*, which is then separated into four quarters: *h*_*NE*_, *h*_*SE*_, *h*_*SW*_, *h*_*NW*_. For example, with filter *h*_*NE *_coefficients, the other three quarters are set to zero, as is shown for the example filters in Figure 1. Due to this choice of separation of the sub-filters, the method might have difficulties detecting objects with complex shapes, e.g. cones or curly objects.

The original image *f *is filtered with the four filters in order to obtain spatial information from four directions around each pixel, giving insight into whether a specific pixel is part of an object or not. The binary output image is obtained by comparing the maximum pixel value from the filtered images to the original pixel value scaled by the factor *α *at each image coordinate (*i, j*). Formally, the binary image bw is defined at pixel location (*i, j*) as(5)

where  is the image filtered with the kernel at direction *NE *(and similarly for the other directions). The filtering directions are illustrated in Figure 1. Hence, the user-definable parameters are the radius *R *which relates to the object size, and the scaling factor *α *which can be used for tuning the segmentation threshold. By using the aforementioned binarization method one can take into account the possibility of non-uniform background, i.e., object presence is decided based on the local features.

#### Local spot enhancement filtering

Local enhancement filtering (LEF) is another novel method for subcellular object detection. LEF is based on a matched filter that enhances spot-like structures and suppresses background intensity. The method starts by scaling the average intensity of the image into a predefined mean, thereby reducing the effect of global intensity differences between images. The square filtering kernel *H *that is used for matched filtering is defined in two parts. First, the inner part is a circular support area  that enhances local intensity peaks. Second, the area in square kernel that is left outside the circular area  is used for suppressing the background by division. Thus, the filtering operation for pixel coordinate (*i*, *j*) can be expressed as follows(6)

where the filtering provides a so-called spot likelihood image  that needs to be thresholded. The thresholding, performed by the product of the sensitivity threshold and the standard deviation of the spot likelihood image (*th*_*s *_× ) provides the final detection result. The division of the kernel area into inner and outer areas is not limited and it could be done based on assumed objects shape, enabling adjustments based on prior knowledge about the objects of interest. In this study, we keep the kernel fixed in order to avoid additional parameter tuning. In this case, the weighting parameter *th*_*s *_for thresholding is the only free parameter.

#### Morphometry based on granulometric analysis

The morphometry method for spot detection, abbreviated here as MGI, is adapted from [23] where automated morphometry was proposed for the quantification of synaptic boutons in neurons. The automated morphometry is based on granulometric analysis. The method first calculates granulometry by using morphology with varying disc sizes *d*, yielding a so-called granulometric index, or size density, *G*(*d*) [23-25]. The granulometric index is then used to select the scale of interest, which in our case involved automatically choosing the two highest peaks in *G*(*d*), denoted as *d*_*low *_and *d*_*high*_. Choosing the scale of interest is critical for the outcome, but for compatibility with high-throughput analysis, we chose to automate the scale selection. The scale of interest is used for constructing the corresponding opening images *I*_*low *_= *I *◦*E*(*d*_*low*_) and *I*_*high *_= *I *◦*E*(*d*_*high*_), where ◦ means grayscale opening and *E *is the disk-shape structuring element. Subtracting *I*_*high *_and *I*_*low*_gives the image where the structures of the desired scale should be present. Further, the structures of interest are extracted by masking with binary image obtained with k-means clustering. Finally, integral thresholding [23] gives the particle detection result within the area that was masked with k-means clustering. Notably, we used our version of the automated morphometry algorithm with default parameters, requiring no parameter tuning.

#### Multiscale product of wavelet coefficients

Detection based on the multiscale product of wavelet coefficients (MW) was presented in [26]. This method extracts bright spots by calculating the products between different support scales of the à trous wavelet transform. Briefly, the algorithm is based on the assumption that, unlike noise or large objects, spots will be present at each scale of the wavelet decomposition, and thus will appear in the multiscale product. The MW method is adapted from [26], where the wavelet representation is obtained as a separable B3-spline wavelet transform by convolving the image *A*_0_(*x, y*) column by column and row by row with a [1/16, 1/4, 3/8, 1/4, 1/16] kernel, resulting in a smoothed image *A*_1_(*x*, *y*). The corresponding wavelet layer is given as *W*_1_(*x*, *y*) = *A*_0_(*x*, *y*) - *A*_1_(*x*, *y*). The convolution is then repeated recursively *J *times, augmenting the kernel at each step *i *by padding 2^*i*-1 ^- 1 zeros between the kernel coefficients. By reaching level *J *in recursion a total of *J *+ 1 images are obtained and are used to construct the wavelet representation *W *= *W*_1_,..., *W*_*J*_, *A*_*J *_of the original image, where *W*_*i*_(*x*, *y*) = *A*_*i*-1_(*x*, *y*)-*A*_*i*_(*x*, *y*), and 1 <*i *<*J*. Spot detection is based on the pixelwise multiscale product of the reconstruction layers *W*_*i*_, defined for pixel position (*x*, *y*) as follows:(7)

where *J *denotes the scales. To repress noise, the wavelet coefficients are thresholded prior to multiplication. Here, we use the hard thresholding scheme proposed in [26], where the threshold is given as 3 × *σ*_*i*_, and *σ*_*i *_is estimated to be MAD(*W*_*i*_)/0.67. The heuristics for choosing the actual objects from the multiscale product include thresholding according to a user-specified detection level. In this study, we use the number of scales *J *and the detection level *l*_*d *_as free parameters.

#### Source extractor

Unlike most filtering methods examined in this study, SourceExtractor (SE) [27] estimates the background in blocks and removes it before filtering with a Gaussian kernel. Background removal is also performed in blocks, the size of which is controlled here by a user-definable parameter. The background estimate is achieved by clipping the intensity histogram at both ends until the histogram converges at three standard deviations around the median. When the standard deviation is changed by less than 20% during the clipping process, the mean is taken to be the background intensity. Otherwise, the background is estimated to be BG = 2.5 × Median - 1.5 × Mean. Pixelwise, the background estimate is then obtained by interpolating the blockwise background estimates.

After filtering, the result is thresholded to provide an initial estimate of the objects. In our implementation, we use two scaling parameters to control the thresholding: *th*_detect _for scaling the standard deviation of background subtracted intensities and *th*_BG _for scaling the background removal. Thus, the thresholding is defined as:(8)

where BG is the estimate for the background, *σ *is standard deviation of the intensity, *f *is the input image, and *bw *gives the binary detection result, each defined here in pixel location (*i*, *j*). By setting *th*_BG _= 0, the version given in [27] is obtained. The detected objects, i.e. the areas in the intensity image under the connected components in the binary image bw, are then processed further in the deblending phase, where possible overlapping sources are separated. Briefly, the deblending proceeds by splitting the detected object into 30 slices inside the intensity range (from the detection threshold to the highest intensity peak) of the object. Starting with the highest intensity peaks, the algorithm takes each slice and determines whether two branches originating from different intensity peaks within the same object should be separated as different objects. The deblending algorithm considers the integrated pixel intensity of the branch relative to the total integrated intensity of the detected object as a basis for determining the separation, as explained in [27].

The original application area of Source Extractor is as far from subcellular object detection as possible; it was designed for analysis of galaxy-survey data [27]. Though the method has been widely applied across many disciplines, to the best of our knowledge, its use in subcellular spot detection has not been reported. The applicability of Source Extractor in the analysis of subcellular structures underscores the generality of the problem of finding bright spots within images.

#### Sub-pixel location detection

The detection method in [10] was used for defining sub-pixel locations (SPL) of single molecules in low SNR (signal-to-noise ratio) images. The detection, though originally intended to be used in tracking, can be used as an independent module for identifying spots. The algorithm detects local intensity maxima by comparing to neighboring pixel intensities and the standard deviation of the local background. In [10], temporal averaging is used to reduce intensity variation prior to detection. However, we omitted the time averaging step since it is only applicable in the context of time-lapse imaging.

The method proceeds as follows. Within a window, the central pixel is chosen to be a potential spot if it is brighter than its surrounding pixels. The initial detection is further controlled by testing against the standard deviation of the local background. A user-defined parameter *α*, the only free parameter for SPL used in this study, controls the local maxima detection. This parameter defines the limit for type I errors in the initial local maxima detection. Sub-pixel locations are estimated for the local maxima that pass the criteria by fitting a 2D Gaussian kernel iteratively as described in [10,28]. Like the feature point detection method, we use SPL only for estimating the locations of detected spots, therefore it can be used to count the number of spots and for object-level comparisons, but not for pixel-level evaluation.

#### Top-hat filtering by grayscale morphological opening

The grayscale morphological top-hat filtering [25,29] acts as a local background removal function, simultaneously enhancing round, spot-like structures. Here we combine top-hat filtering and automated thresholding to form a spot detection method, abbreviated as THE. Essentially, the filtering phase performs grayscale opening with a flat disk-shaped structuring element *E *of radius *r *and subtracts it from the original image *f*. More formally, the top-hat filtering result is given as *f*_diff _= *f *- *f *◦ *E*(*r*), where ◦ denotes grayscale opening. In the filtering result, the objects roughly of size determined by *r *should be enhanced, and background removed.

The resulting image *f*_diff _needs to be thresholded in order to obtain a binary mask for spots. We tested several histogram-based segmentation methods [30,31], and applied an entropy-based thresholding [32] which produced slightly more conservative values for images with spots than many other thresholding methods. Thus, instead of parameterizing the detection threshold or applying any post-segmentation constraints, we use top-hat filtering in a more automated manner, which requires considerably less parameter tuning.

### Data

#### Simulated experiments

The most natural way of comparing segmentation algorithms is by a pixelwise comparison. However, constructing a reference segmentation in which all of the pixels belong to biologically meaningful small spots would be difficult. Creating a reliable and representative reference result is difficult because, on the one hand, it is extremely tedious to manually analyze a large number of spots in a reliable manner, and on the other hand, analyzing a relatively small number of spots is statistically inadequate. Thus, to enable pixelwise comparisons against a reference result, we used simulated experiments published previously as a benchmark set in [12].

The simulated image set, generated by using the SIMCEP cell image simulation framework [33,34], consists of 20 images with nuclei, cytoplasmic areas, and subcellular objects each having their own channel in the RGB image. Noise, i.e. intensity variations in cell texture, and blurring for out-of-focus objects are also introduced in the simulation process [34] in order to give the simulated images some level of error akin to that encountered in experimentally derived images. Prior to the analysis, the images are converted to grayscale using the standard conversion of 0.2989 × R + 0.5870 × G + 0.1140 × B. After this conversion, subcellular objects in the grayscale images have slightly higher intensities than their surroundings.

#### Frames from image stacks of yeast cells

The second data set contains frames from image stacks obtained with wide-field imaging. The objects are P-bodies, visualized by Edc3 protein fused to green fluorescent protein (using a strain created by Huh, et al., [35]). Stacks of 28 frames along the z-axis (every 0.3 *μ*m) were acquired using a Leica DMGI 6000B microscope equipped with motorized X-Y stage, a high quantum efficiency cooled back-illuminated Qimaging Rolera-MGi CCD camera, and integrated software control (BD Bioscience Bioimaging IPLab). The images were acquired under oil using a 63× objective/1.40 NA Plan APO oil lens.

Stacks usually consist of a set of frames starting with images in which the objects of interest are not yet in focus. As the microscope scans through the sample in the Z plane, objects come in focus, appear in a set of frames, and then vanish as the scanning proceeds past the object's focus area. The implication for the analysis task is that the spot detection methods must be able to detect objects only in the in-focus frames. We selected seven stacks of images and from each stack chose four frames such that one frame is empty (only out-of-focus objects are in the image and no P-bodies are marked in the reference result) and three frames with varying number of P-bodies that are present and in focus. In total, 28 frames are used in this study.

For the selected frames a reference result was manually determined by two observers. In order to limit the number of comparisons we chose to combine the results from two observers such that we included all spots in our reference. Due to the small spot size and the noisy appearance of the wide-field microscope image, the objects are marked with a fixed-size spot but the area is not outlined in detail. As a consequence, the manual reference result can be used for object level comparison, i.e. to assess whether an object is found or not, but not as a pixel-level ground truth result.

#### Well plate experiments of a human osteosarcoma cell line

A major application of bright spot detection of subcellular organelles is in high-throughput screening, where for example the effects of gene knock-outs or differences in responses to varying dose levels of a particular stimulus are of interest. To test the ability of the various methods to detect differences in populations of cells stimulated with different doses of a drug, we used the SBS CompuCyte Transfluor image set provided by Dr. Ilya Ravkin and available from the Broad Bioimage Benchmark Collection [13]. The images are of a human osteosarcoma cell line. The image set consists of a portion of a 96-well plate containing 3 replica rows and 12 different concentrations of isoproterenol. Importantly, stimulation with different doses of isoproterenol affects the appearance of small vesicle-like spots. There are four acquired fields per well, resulting in 144 total images. The image set also contains specific staining for nuclei, which we used to determine the number of cells per image. Thus, the outcome of the analysis is an assessment of the average number of vesicles per cell in each image, with the images grouped by dose level. These results can be used for indirectly comparing different methods, since no ground truth information for the vesicles is available.

### Performance evaluation metrics

Several metrics for performance evaluation exist when reference result, for example object number and locations are known [36,37]. For measuring the accuracy of detection algorithms, we chose the following commonly applied metrics.

First, a true positive (TP) is defined as a correctly found object, and a false positive (FP) is a detected object for which there is no match in the reference image. A false negative (FN) corresponds to a missing object in the detection result. The same definitions may also be applied for pixel-level analysis. In accordance with [36], we define precision *p *(also noted as positive predictive value) as(9)

and recall *r *(also noted as sensitivity) as(10)

By intuition, detecting objects where no true objects exist is penalized in *p*, whereas failure to detect true objects is penalized in *r*.

Furthermore, the F-score can be obtained as a harmonic mean of precision and recall [36],(11)

The F-score combines precision and recall as a single measure of segmentation accuracy, making it a useful parameter for evaluation purposes.

## Results and Discussion

### Parameter optimization

**Figure 2:**
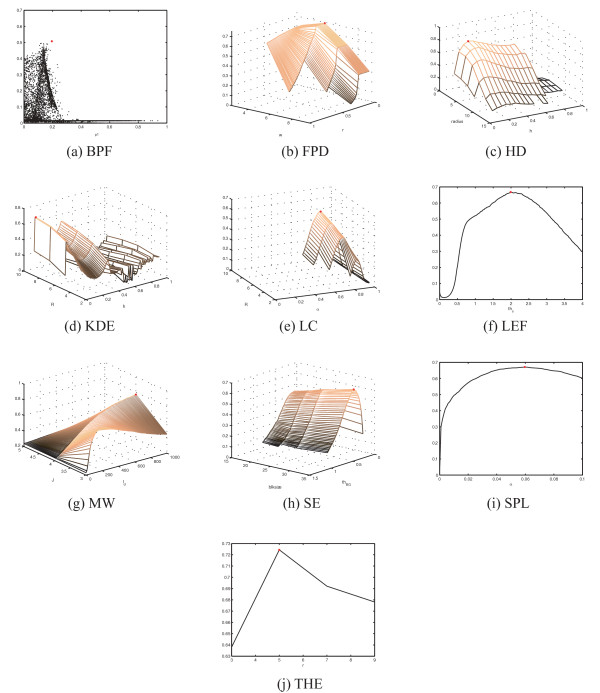
**Parameter tuning for spot detection methods was performed in exhaustive grid search manner using F-score as the measure of detection accuracy**. The optimal settings within the search space (yielding maximum F-score) are shown with a red dot.

**Figure 3:**
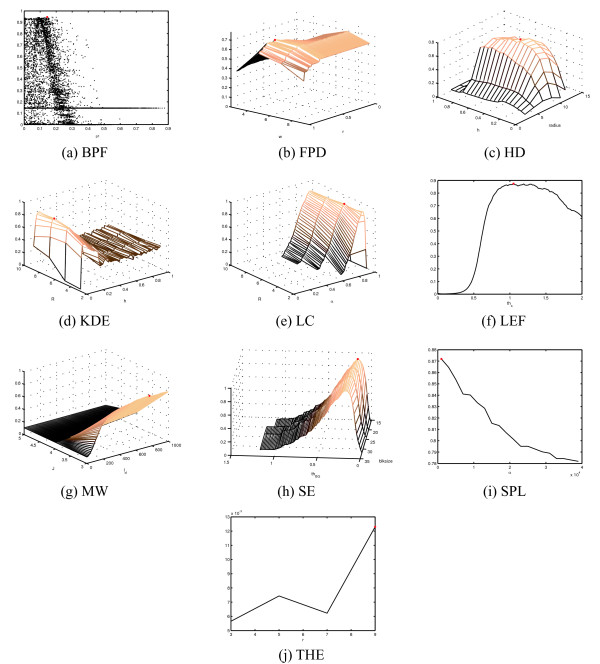
**Parameter tuning results for yeast image stacks**. Red dot denotes the result with optimal parameter settings within the search space for the applied data.

Many of the detection methods proposed in the literature incorporate one or more parameters which can be tuned to enable detection in different situations. The methods proposed in this study also require user-defined values for input parameters. In spot detection, parameters typically provide information about object size (e.g. LC, BPF, THE, HD) and probability (FPD), permit tuning of the detection threshold (LC, KDE, LEF), or specify the applied option within a family of methods (such as in KDE). Because the parameter values have a significant effect on the detection accuracy and need to be tuned specifically for the applied data, we performed parameter optimization for the two datasets with ground truth references and recall *r *(also noted as sensitivity) as by sampling the parameter space in a grid search manner. By using the F-score described in Equation 11 as a measure of detecting performance, the grid-search can be used to tune the parameters optimally within the search space for the applied data. The results of parameter tuning for the simulated data and for yeast image stack data are shown in Figure 2 and Figure 3, respectively. We note that for methods with more that two free parameters, we have chosen two for visualization purposes, and a grid search was carried out to identify the optimal combination of the remaining parameters to construct the images shown. The detection accuracies as a function of all free parameters can be found in the supplementary materials. To facilitate an objective comparison of these methods, we used of a common measure (F-score), which is optimized within the parameter ranges. The results obtained represent the best possible result within the input parameter space. The parameter tuning results in Figure 2 and 3 also provide information about the sensitivity to changes in parameter values, which may be useful when tuning methods to new data. The grid search approach also solves the difficult problem of parameter tuning, with the cost of exhaustive computations requiring large amounts of processor time. Although parameter tuning can be accomplished by performing the calculations in parallel on a grid-computer network (as was done in this study), the problem of setting the value range and sampling the parameters remains. For some parameters, such as those related to object size, reasonable value ranges may be set intuitively. The fact that many parameters are natural numbers makes the process easier. However, real-valued parameters, such as probabilities and tuning factors need to be sampled more densely and their dynamics is less predictable. For example, the size of the structuring element can be defined by testing with a few values (Figure 2i and 3i), whereas the significance value *α *needs to be sampled more densely (Figure 2h and 3h). Furthermore, parameter sampling when parameters depend on each other becomes even more challenging. As an example, the parameters of BPF defining the pass band need to be in increasing order, leading to sparse point-cloud type sampling (Figure 2a and 3a) instead of a smooth curve or surface.

Allowing the user to tune many parameters leads to a highly adaptable method, but also requires considerable effort to ensure reasonable (or ideally optimal) performance. Thus, the calculation times for the optimization procedures vary greatly between methods. While a detailed discussion of the effect of each parameter for the 11 methods is beyond the scope of this article, we offer a list of parameters for each method along with the applied ranges in the supplementary materials. Lastly, we note that by tuning different parameters than the ones in this study, and by adding more parameters to the methods, the methods may be further customized for analysis tasks.

### Results for simulated images with pixel-level reference

First, we consider the validation of algorithm performance with simulated images in which the noise-level and other image characteristics are known. In this case, we calculated the number of objects detected in the 20 images by each method and determined whether the differences between object counts were significant, using the non-parametric Kruskal-Wallis test of whether the medians of multiple groups are equal. Our results suggest that detection results do infact differ significantly (*p *~ 0 while *p *< 0.01 was considered statistically significant). Wilcoxon rank sum tests between result pairs further support this claim by showing that most of the results do not have the same median.

**Figure 4:**
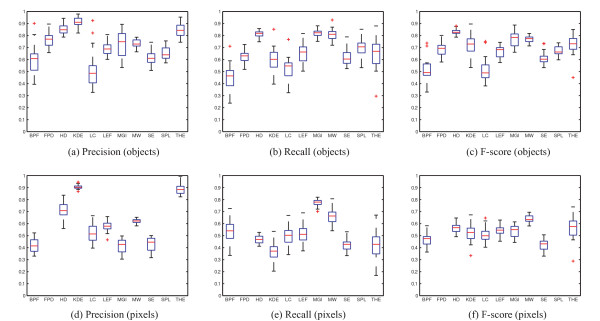
**Precision, recall, and F-score are calculated using computer-generated ground truth as reference at object level (a-c) and pixel level (d-f)**. Note that the two algorithms (FPD and SPL) are omitted from pixel-level comparison in d-f.

For synthetic images the comparison can be made at both the pixel-level, which is perhaps the most natural way of defining segmentation accuracy, and at the object level, as was done for the wide-field microscope images. The object level comparison for the set of 20 simulated images is presented in Figure 4a-c, and the corresponding pixelwise comparison is presented in Figure 4d-f. We note that two of the methods, namely FPD and SPL, cannot be included in pixel-level comparison because they were used for estimating spot locations instead of segmenting objects. The results in this article are calculated for the low quality simulated image set (described in [12]).

**Figure 5:**
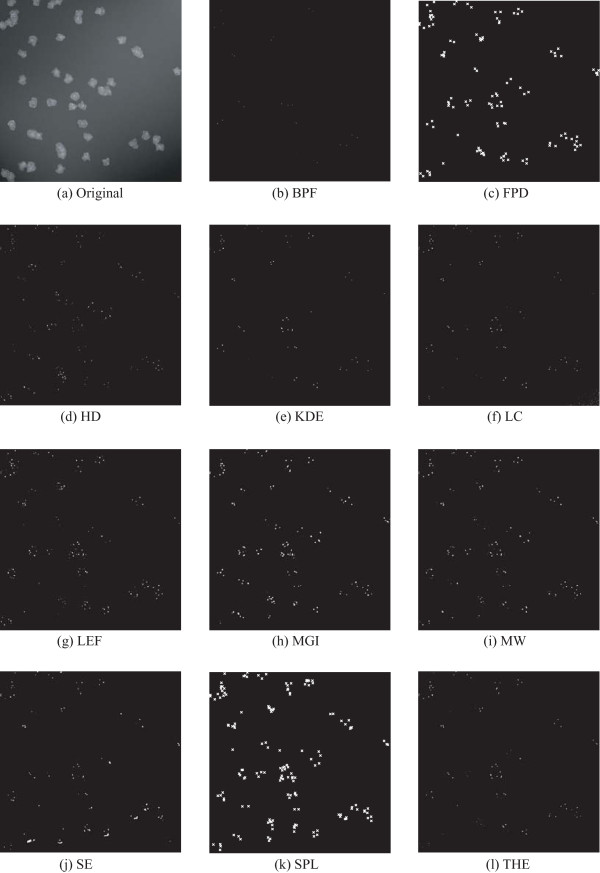
**Examples of detection results for a simulated image**. Note that for FDP and SPL, the detection has been visualized as a cross centered in the detected point. For others, the result is shown as a binary segmentation mask.

In the pixel-level comparison none of the methods stand out with superior accuracy, although MW received a slightly better F-score value than the other algorithms. For object-level comparison, HD had the most accurate F-score, followed by MGI and MW. In considering the relationship between method performance at the pixel and object levels, clearly there is some level of correlation between the two sets of results. Intuition would suggest that it is easier to merely detect an object than it is to define its area accurately. Consistent with this view, the pixelwise results are generally lower than the object level results. The relatively subtle differences in the pixel-level results Figure 4d-f do not allow identification of a single algorithm that would be superior in both categories. For example, HD seems to find the objects well, but its performance in pixelwise comparison does not stand out from others. Conversely, the accuracy of LC is lower than average for object detection, but its performance in pixel level is close to the average accuracy. Examples of segmentation results are shown in Figure 5.

### Results for yeast images with object level reference

After the analysis of the simulated images, we considered subcellular detection in wide-field images. Wide-field microscope images of yeast P-bodies give insight into algorithm performance in the context of actual experimentally derived data. In these images noise and contrast limit the detection accuracy, but objects are well scattered, and the object count per image is relatively low. We used the manually constructed reference images and the performance measures given in Eqs. 9-11 to numerically compare the algorithms. The performance metrics were calculated for a set of 28 images containing a total of 262 objects, and the results are listed in Table 2.

**Table 2:** Results for yeast image frames

Algorithm	precision	recall	F-score
BPF	0.9570	0.9351	0.9459
FPD	0.5964	0.8969	0.7165
HD	0.8682	0.7290	0.7925
KDE	0.9116	0.8664	0.8885
LC	0.9396	0.9504	0.9450
LEF	0.8712	0.8779	0.8745
MGI	0.6175	0.8626	0.7198
MW	0.7645	0.8550	0.8072
SE	0.9318	0.9389	0.9354
SPL	0.8167	0.9351	0.8719
THE	0.0062	0.9733	0.0123

Summary of numerical results for the frames from image stacks of yeast cells. The reported results are the precision, recall, and F-score values calculated for 28 frames, seven out of which had no objects according to manual analysis. The results are the maximum F-scores obtained in parameter tuning by grid search, i.e., the F-score shown with red dots in Fig. 3.

**Figure 6:**
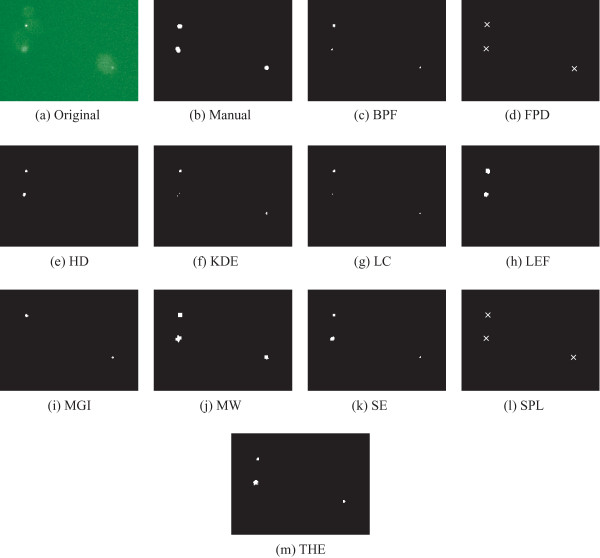
**Example frame of yeast P-body image stacks and detection results by the algorithms**. Manually marked objects in reference result are also shown for the same area. Note that the objects detected by FPD and SPL are illustrated as crosses whereas the actual segmentation results are shown for other algorithms. Note that the parameters of different methods are tuned for the whole dataset, not for this particular image. The original image has been enhanced for illustration purposes.

We compared the precision value, which penalizes extra detections and the recall value, which penalizes missed objects. With the exception of THE, most methods produced sufficiently accurate results, as evaluated by F-score (Table 2). Within that set of accurate methods, BPF, LC, and SE provided the best results, and KDE, LEF, and SPL (with F-scores close to 0.9) were the next most satisfactory. In contrast, the precision results reveal clear differences that require further attention. The precision of the THE, MGI and FPD methods stand out as having significantly high variance. Inspection of the segmentation results reveals that the poor performance of all three is due to their performance in the empty images, i.e. images with no objects located in manual analysis. In these cases, false positive detections in empty images lead to low precision. The majority of the images have in-focus P-bodies, and for those images THE, FPD and MGI gave reasonable results. With respect to the recall values, THE is the highest while both FPD and MGI also score well. Examples of detection results are shown in Figure 6, where a zoomed area in a single frame and the corresponding reference result are shown together with the detection results produced by all eleven algorithms.

### Results for osteosarcoma well plate images

**Figure 7:**
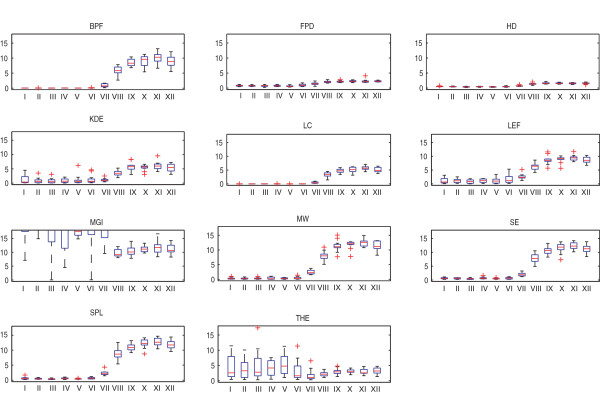
**Results for the well plate experiment**. The boxplots show number of spots detected per cell in each image, grouped according to the dose level. Parameters of each detection method have been tuned for the data, but here the lack of ground truth reference renders parameter optimization through comparison against reference impossible.

**Figure 8:**
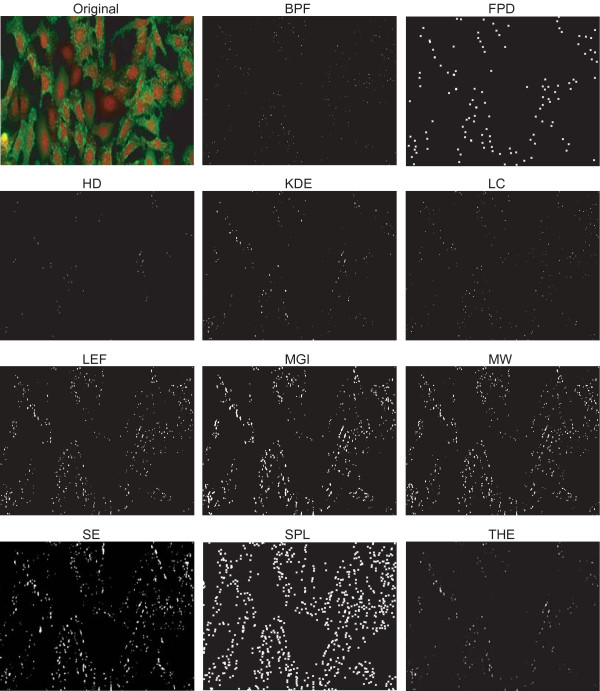
**Example of well plate images and detection results by eleven algorithms**. Note that the objects detected by FPD and SPL are illustrated as crosses whereas the actual segmentation results are shown for other algorithms. The original image has been contrast and intensity enhanced for illustration purposes.

Next, we considered the analysis of well plate experiments as an example of image-based high-throughput measurements. High-throughput experiments typically challenge image analysis with high object density, high levels of background staining, and high variation of image characteristics across the experiment. The images used for our analysis contain cell populations that are expected respond to a given dose of a drug with varying levels of vesicle-like structures. We obtained an estimate of the average number of vesicles per cell in each image by calculating the number of vesicles in all 144 images and dividing the number of vesicles by the number of cells in each image. We then grouped the results by the reported dose level. The result is a measure of the dose responses for the cell populations determined by each of the eleven algorithms. We then used these dose responses as an indirect comparison between the detection algorithms. The results are shown in Figure 7, and a sample image with corresponding detection results is shown in Figure 8.

The dose responses in Figure 7 form a step-like pattern, with very few vesicles per cell in low-dose populations (dose levels I to VI), increasing vesicle numbers beginning with dose level VII and peak vesicle numbers at dose level XI. Increasing the dosage beyond that of level XI (i.e. level XII) does not appear to increase the average number of vesicle structures per cell. This behavior is consistent with saturation as the dose concentration increases.

Although there are differences in the absolute number of vesicles per cell in low dose images and the magnitude of the difference between the low and high dose images, all methods (except MGI and THE) produce this step-like dose response curve. For example, the step given by FPD, LC, and KDE is substantially lower than those by BPF, SE, and SPL. The result given by MGI and THE resemble the others for the high dose values where the images contain a large number of vesicles. When vesicles are few in number or not present at all, the methods give false detections. The clear differences in the dose responses obtained with different algorithms suggests that any downstream analysis, such as clustering or classification of populations based on the vesicle counts could produce significantly different results.

### Comparison of relative similarities

**Figure 9:**
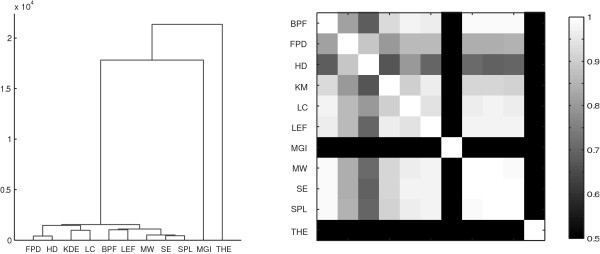
**Dendrogram and pairwise correlation coefficients calculated based on object counts for all three image sets show similarities between algorithms**. Correlation values lower than 0.5 are shown as black in the figure.

To further explore the results (i.e. the number of objects detected across all images) obtained for all three image sets, we preformed hierarchical clustering and visualized the results as a dendrogram (Figure 9). Figure 9 illustrates the extent of the similarity between some of the algorithms across the set of close to 200 images, with FPD and HD being the closest matches and SE, MW, and SPL also forming a tight cluster. Some of the closest matches include algorithms that have similar detection principles. For example, both SE and SPL use matching of a Gaussian kernel into local maxima as their backbone, SE by filtering into a background subtracted image and SPL by repeated fitting into a local maximum point. However, similar results were also obtained by algorithms with different approaches, e.g. HD and FPD. The dendrogram also identifies methods whose results are significantly different from those obtained by the other methods. For example, both MGI and THE use a morphology-driven detection strategy with automated thresholding. This detection approach is different from that of the filtering-based methods, and as the results in Figure 7 and Table 2 show, these methods perform poorly on empty images when used with a completely automated thresholding as has been done here. In contrast, MGI and THE did perform fairly accurately on the simulated images. Thus, the use of these two methods may be warranted under conditions other than those in which the majority of the other methods perform well.

Finally, we calculated the pairwise correlation coefficients between the object counts obtained for all images. The pairwise correlation values between methods are shown in Figure 9. These correlation results further support the clustering results, namely that results given by MGI and THE differ significantly from the others (low correlation with other results) whereas SE, SPL and MW performed similarly (correlation *>*0.99). The object counts for all image sets that were used for constructing the dendrogram and calculating the correlations are available at the supplementary site.

## Conclusions

We have studied the performance of eleven subcellular object detection algorithms under different analysis scenarios. Our study included real images of high-throughput well plate experiments for indirect comparison of the algorithms, as well as frames from image stacks of yeast P-bodies for which the object-level reference information was available. In addition, we used simulated images with small subcellular objects, thereby enabling a pixel-level comparison of algorithms against a computer-generated ground truth.

Results for the simulated images gave detailed insight into the performance of the methods. In the simulated image set all the images had the same number of subcellular objects. The object counts obtained for the image set revealed that statistically significant differences exist between the algorithms. The small spots proved to be rather difficult to detect, highest object level accuracy (in terms of F-score) being 0.8249 given by HD. Worth noting is that also MGI (0.7698), THE (0.7244), and FPD (0.6905) all perform well for the set where all images had spots, all of which had problems with the empty images. Simulation allowed also pixel-level comparison, where MW gave the most accurate segmentation by a slight difference when measured by F-score, but none of the methods provided outstanding accuracy. The pixel-level results confirm how challenging it is to accurately segment small particles in noisy and partially blurred images with heavy background fluorescence. Moreover, the limitations in segmentation performance on pixel-level raise a question about the reliability of shape, size or morphology features extracted from subcellular objects in standard fluorescence microscopy measurements.

Second, the high level of F-score values for the detection of GFP labeled P-bodies in wide-field microscope images proved that all methods can be used for accurate detection of bright spots when background intensity is on a moderate level. The highest F-scores between manually located reference result and automated analysis result were given by BPF (0.9459), LC (0.9450), and SE (0.9354). Furthermore, FPD, MGI, and THE had difficulties in the handling of empty frames of wide-field microscope images, which shows as a high number of false positives leading to low precision values. Excluding the empty frames, all these three algorithms were accurate for the frames with in-focus P-bodies, which is confirmed by the high recall values.

Third, results for the human osteosarcoma well plate measurement data further confirmed how some of the algorithms failed to cope with a large data set where images contain varying amount of small spots. Examples of poor handling of varying conditions were THE and MGI algorithms. Our implementation of MGI detects automatically the scale of interest, and in case of no or very few vesicles it fits to the scale of cells. The implementation of THE included automated thresholding, which in this case assumed the data to include two groups: objects and background. Thus, neither of these two algorithms was able to handle all images with same parameter settings when used in the way described here. However, including free parameters for tuning the segmentation or preprocessing steps could lead to better results. Also FPD and HD produced less obvious ramp as a dose response. FPD assumes certain level of spots to be present in all images (percentile-based detection), which explains why varying probability for spots within experiment may cause problems in detection. HD method, despite its heavy parametrization, seems to make a compromise where very few false objects are detected, but also part of true objects are missed. Apart from MGI and THE, all of the compared algorithms produced a step-like dose response, suggesting that the methods can be used for detecting differences between populations exposed under varying levels of stimulus. The results given by the 11 algorithms confirm that they all are very useful in spot detection tasks, but the results also show clear differences in terms of their ability to detect small, vesicle-like objects and to adjust to varying conditions. The handling of images containing very few, if any, small spots, in particular, brought out significant differences between the algorithms. Since handling such images can be fundamental for some applications, the algorithms should be chosen with care.

Finally, some remarks on the performance of the three detection algorithms originally developed for tracking purposes, i.e. FPD, HD, and SPL. Though developed for similar purposes, the methods have different approaches for detection. By definition, FPD tends to detect roughly the same number of objects for a set of images when used with fixed parameter settings. From a tracking point of view, this is a reasonable assumption if the number of particles is expected to be rather constant throughout the imaging sequence. The results for simulated image set support this conclusion, since the the number of particles stays constant in this set. On the other hand, when the number of particles present in the images changes dramatically over the course of the imaging period, the HD and SPL approaches are likely to give more accurate performance. This assertion is supported by our results. SPL adjusts well to varying conditions in well plate and wide-field images, and HD performs reasonably well in varying conditions, avoiding excessive false positive detections for empty yeast images (high precision value), though doing so at the cost of missing some spots (low recall value). We note that for the yeast stack images, even tuning the five free parameters of HD did not provide results as accurate as those with the single open parameter of SPL. For the simulated images, on the other hand, HD gave the most accurate results among all 11 algorithms, outperforming both FPD and SPL. Although detection accuracy does not directly predict subsequent tracking performance, choosing a method based on careful testing may be beneficial.

Thus, although detection algorithms are problem-specific, the systematic comparison of methods with a large set of test images can help choose the best method for the particular imaging challenge. Using a systematic approach, algorithms can be compared under varying conditions, providing useful information for various use cases. Our study also makes use of recently published benchmark datasets in order to evaluate algorithms. Importantly, evaluation based on a wide range of images tests the algorithms with an objective framework in which performance has not been tuned for a small set of images with specific characteristics. For example, subcellular object detection in modern high-throughput imaging experiments provides a challenge for image analysis because contrast, intensity, and number of spots may vary significantly within the same experiment. Systematic testing of algorithm performance with large image sets, as was done in this study, allows one to predict algorithm performance in such tasks. Supplementary material, including additional result figures and an algorithm toolbox as a CellProfiler [38] compatible module written in MATLAB (The MathWorks, Natick, MA) are available for download at http://www.cs.tut.fi/sgn/csb/subcell.

## Authors' contributions

PR planned the study, and wrote the manuscript. PR and TÄ carried out experimental calculations. TÄ, SC, PR, and JS were responsible of algorithm implementations. CG-T carried out experimental work related to yeast imaging, and CG-T & JS performed manual analysis for the yeast image set. TÄ, SC, CG-T, JS, MB and AMD helped in manuscript editing. MB and LP participated in design of the study. AMD supervised the experimental work with yeast. LP and OY-H conceived of the study. All authors read and approved the final manuscript.
